# Thrombophlebitis Migrans (Trousseau Syndrome) in Pancreatic Adenocarcinoma: An Autopsy Report

**DOI:** 10.7759/cureus.5528

**Published:** 2019-08-30

**Authors:** George S Stoyanov, Deyan L Dzhenkov, Maria Tzaneva

**Affiliations:** 1 Pathology, Medical University of Varna, Varna, BGR; 2 General and Clinical Pathology, Forensic Medicine and Deontology, Medical University of Varna, Varna, BGR

**Keywords:** thrombophlebitis migrans, trousseau syndrome, pancreatic adenocarcinoma

## Abstract

First described by a French physician Armand Trousseau, the Trousseau sign of malignancy is a classic example of paraneoplastic syndrome, caused by adenocarcinomas predominantly of the stomach, pancreas, and lung. The condition presents as recurring and migrating episodes of thrombophlebitis that can involve the upper and lower limbs, thoracic and abdominal wall, and the major blood vessels of the abdomen. These recurring episodes may lead to a detachment of a thrombus and the formation of pulmonary thromboembolism (PTE), often proving fatal for the patient. Herein we present a case of a 60-year-old male patient referred for autopsy. The patient was admitted with acute onset of gastrointestinal tract symptoms and after admission, his condition deteriorated rapidly, with new-onset neurological symptoms and an acute massive fatal episode of PTE. Previous medical history was uneventful, apart from several episodes of recurring lower limb thrombophlebitis for the past six months, resulting in two prior episodes of PTE. The autopsy revealed a massive PTE, with multiple thrombi in the venous vessels, including the two common iliac veins and the inferior vena cava. Histological evaluation revealed pancreatic adenocarcinoma with distant metastasis to a number of organs and abdominal thrombophlebitis with embolization of the pulmonary arteries.

## Introduction

Trousseau syndrome, also commonly referred to as Trousseau’s sign of malignancy to avoid confusion with Trousseau’s sign of latent tetany is a type of paraneoplastic syndrome caused by adenocarcinomas, predominantly gastric, pancreatic and pulmonary, presenting as recurrent and migrating episodes of thrombophlebitis [1-3]. Thrombophlebitis may arise in typical areas such as the lower limbs, but can also disappear and reappear in a different location, hence migratory, such as the abdominal and thoracic wall, abdominal blood vessels, upper limbs, and neck [2].

The pathophysiology of the phenomena is not well established, but the mucin compounds produced by adenocarcinomas have been found to interact with the selectin family of adhesion molecules, leading to thrombocyte activation and aggregation [2, 4].

These recurring episodes often result in detachment of thrombi and distant embolization, predominantly pulmonary thromboembolism (PTE); however, if the emboli persists in the venous system and traverse a patient foramen ovale, they can enter the systemic circulation, presenting as paradoxical cerebral embolism.

## Case presentation

A 60-year-old male patient was referred for autopsy in the General and Clinical Pathology department of the St. Marina University Hospital, Varna, Bulgaria. The patient was admitted to the Infectious Disease department with acute onset of gastrointestinal tract symptoms-lack of appetite, debilitating physical weakness, and multiple watery stools. After admission, the patient’s condition deteriorated rapidly, with new-onset neurological symptoms and an acute massive episode of PTE was registered on an electrocardiogram monitor prior to the patient’s death.

Previous medical history was uneventful, apart from several episodes of recurring lower limb thrombophlebitis for the past six months, treated conservatively, resulting in two prior episodes of PTE.

Upon autopsy, a massive PTE was established, with multiple thrombi in the venous vessels, including the two common iliac arteries and vena cava inferior.

The gastrointestinal tract showed severe gas dilatation of the small intestine and colon with hyperemia in the mucosa across the stomach, small, and large intestine. The pancreas had severe gross changes suggestive of pancreatic adenocarcinoma-irregular borders and adhesion to surrounding tissues, solid and grayish-white in color.

The central nervous system section revealed a massive hemorrhage on the left cerebral hemisphere and a lesion, measuring 2/2.4 cm in the left occipital lobe, sharply demarcated from the surrounding brain tissue.

Other gross changes included several thickened zones in the peritoneum, pleura and pericardium, and more than 20 calculi in the urinary bladder.

**Figure 1:**
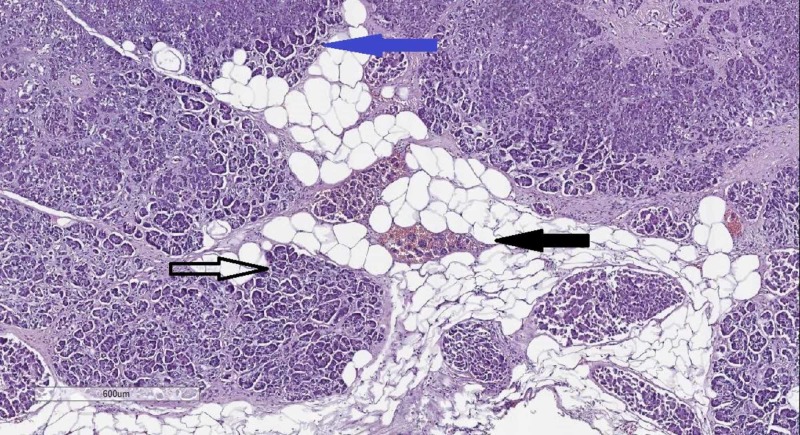
Pancreas histology–a proliferation of atypical glandular structures (hollow arrow), vascular invasion (black arrow), and invasion into the adiposa (blue arrow). Hematoxylin and eosin stain, original magnification 40x

**Figure 2:**
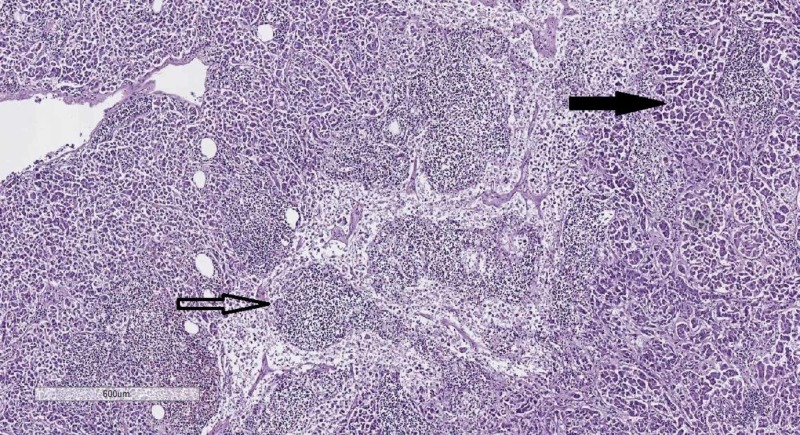
Lymph node histology–remnants of lymph follicles (hollow arrow) and metastasis from atypical glandular structures, identical to those in the pancreas (black arrow). Hematoxylin and eosin stain, original magnification 40x

Histology from the pancreas revealed a proliferation of atypical glandular structures, with invasion into vascular structures and the surrounding adiposa and metastasis to regional lymph nodes (Figures 1-2).

**Figure 3:**
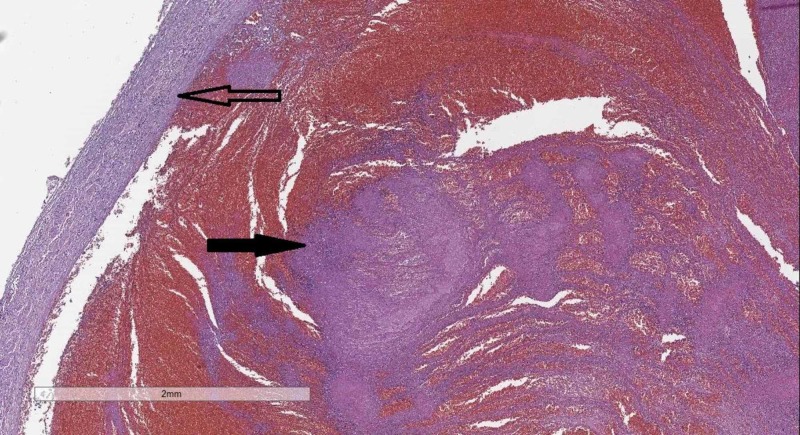
Vena illiaca communis–wall (hollow arrow) and mixed thrombus in the lumen (black arrow). Hematoxylin and eosin stain, original magnification 20x

Histology from the venous vessels of the abdomen showed an abundance of mixed thrombi (Figure 3).

**Figure 4:**
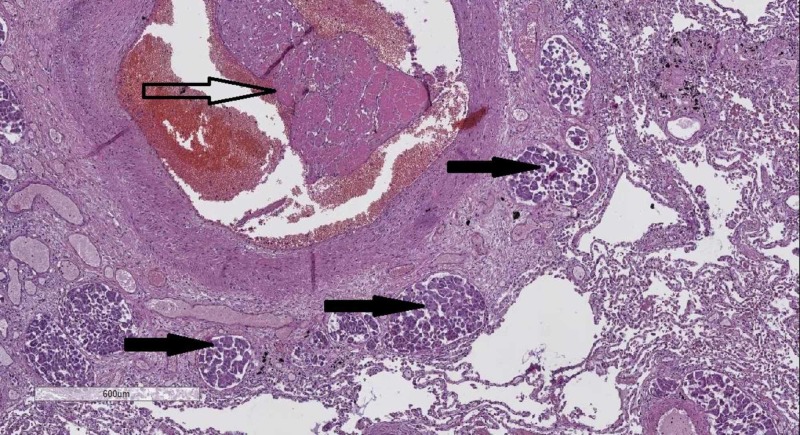
Lung histology–organized thrombotic (hollow arrow) and multiple cellular (black arrow) emboli. Hematoxylin and eosin stain, original magnification 40x

Histology of the lungs revealed an abundance of thrombi, some with organization, and cellular emboli in the vascular tree (Figure 4).

**Figure 5:**
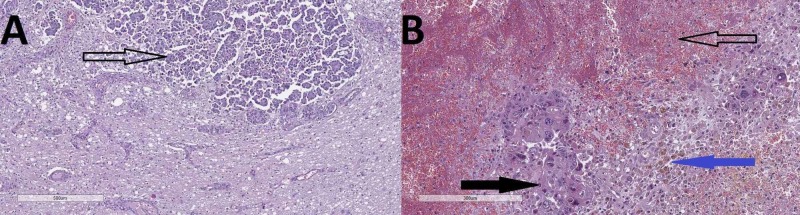
Histology of the brain lesions. Occipital lesion (A)–metastasis from the pancreatic adenocarcinoma (hollow arrow), hematoxylin and eosin stain, original magnification 50x. Grossly hemorrhagic lesion (B)–extravagated erythrocytes (hollow arrow), atypical cells (black arrow) and siderophages (blue arrow), hematoxylin and eosin stain, original magnification 100x.

Histology of the two lesions from brain revealed metastasis from the pancreatic adenocarcinoma, with the grossly hemorrhagic one including siderophages, showing a chronic bleeding nature to it (Figure 5).

**Figure 6:**
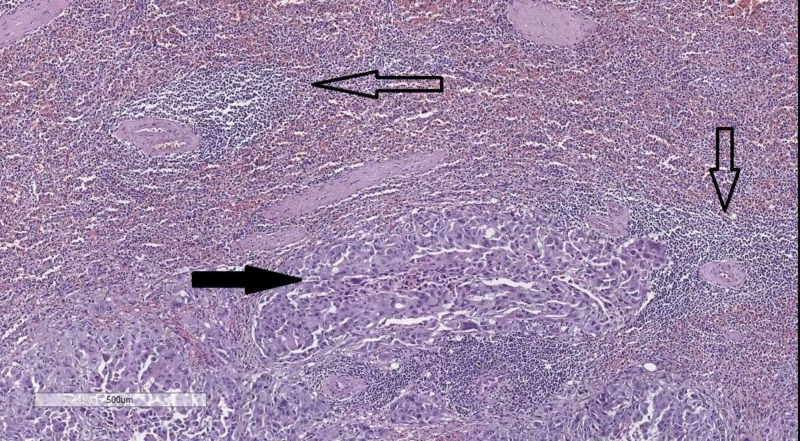
Spleen histology–remnant follicles (hollow arrows) and metastasis from atypical glandular structures, identical to those in the pancreas (black arrow). Hematoxylin and eosin stain, original magnification 50x

Other sites involved from either tumor emboli or metastasis were the pericardium, liver, kidneys, adrenal glands, and spleen (Figure 6).

## Discussion

Trousseau’s sign of malignancy was first described by a French physician Armand Trousseau (born 14 October 1801, Trous, France-died 23 June 1867, Paris, France), amidst his many contributions to modern medicine including Trousseau’s sign of latent tetany, Trousseau-Lallemand bodies (now referred to as Bence-Jones proteins) [3]. He considered the presence of migrating thrombophlebitis nearly pathognomonic for the presence of malignancy, so much so that he diagnosed himself with the condition he first described, which led to him being diagnosed and subsequently dying of gastric adenocarcinoma [3].

A classic example of paraneoplastic syndrome, predominantly in gastric, pancreatic and pulmonary adenocarcinomas, Trousseau’s sign of malignancy has largely lost its clinical value due to improved diagnostic processes [5]. Relatively easy to diagnose clinically, when the patient is followed up, with migration to superficial structures, the condition can lead the physician to think about malignancy.

However, in cases, as the one described, where there are recurring episodes of lower limb thrombophlebitis, migrating to the abdominal blood vessels, the diagnosis is difficult. Furthermore, when thrombophlebitis involves the venous system of the gastrointestinal tract, as in the case described, the clinical picture may be dominated by symptoms of the respective system and further sway the physician away from the correct diagnosis.

## Conclusions

The presented case shows a somewhat atypical presentation of Trousseau’s sign of malignancy, with recurrent lower limb thrombophlebitis migrating to the abdominal vasculature, on the background of advanced pancreatic adenocarcinoma, without any further symptoms. The value of extended clinical examinations in patients with recurrent PTE and thrombophlebitis to exclude any underlining causes, not only malignant, is a key providing adequate levels of healthcare and the prevention of further, possibly lethal, PTE episodes.
